# The INVEST trial: a randomised feasibility trial of psychologically informed vestibular rehabilitation versus current gold standard physiotherapy for people with Persistent Postural Perceptual Dizziness

**DOI:** 10.1007/s00415-022-11107-w

**Published:** 2022-04-10

**Authors:** David Herdman, Sam Norton, Louisa Murdin, Kate Frost, Marousa Pavlou, Rona Moss-Morris

**Affiliations:** 1 Health Psychology, Institute of Psychiatry Psychology and Neuroscience, King’s College London, Guy’s Hospital Campus, Great Maze Pond, London, SE1 9RT UK; 2 St George’s University Hospitals NHS Foundation Trust, London, UK; 3 Centre for Rheumatic Diseases, Faculty of Life Sciences and Medicine, King’s College London, London, UK; 4 Guy’s and St, Thomas’ NHS Foundation Trust, London, UK; 5 Ear Institute, University College London, London, UK; 6 Centre of Human and Applied Physiological Sciences, King’s College London, London, UK

**Keywords:** Vestibular rehabilitation, PPPD, Cognitive behavioral therapy, Dizziness, Vestibular, Feasibility

## Abstract

**Background:**

Persistent postural perceptual dizziness (PPPD) is a common and disabling functional neuro-vestibular disorder. We aimed to determine the feasibility and acceptability of conducting a randomised controlled trial of cognitive-behavioural therapy informed vestibular rehabilitation (INVEST intervention) designed for persistent dizziness.

**Methods:**

A two-armed parallel groups randomised feasibility study of INVEST vs. a time-matched gold standard vestibular rehabilitation (VRT) control. Participants with PPPD were recruited from a specialist vestibular clinic in London, UK. Participants were individually randomised using a minimisation procedure with allocation concealment. Measures of feasibility and clinical outcome were collected and assessed at 4 months.

**Results:**

Forty adults with PPPD were randomised to six sessions of INVEST (*n* = 20) or gold standard VRT (*n* = 20). Overall, 59% of patients screened met the inclusion criteria, of which 80% enrolled. Acceptability of INVEST, as assessed against the theoretical framework of acceptability (TFA), was excellent and 80% adhered to all 6 sessions. There were small to moderate treatment effects in favour of INVEST across all measures, including dizziness handicap, negative illness perceptions, symptom focussing, fear avoidance, and distress (standardised mean difference [SMD]_g_ = 0.45; SMD_g_ = 0.77; SMD_g_ = 0.56; SMD_g_ = 0.50, respectively). No intervention-related serious adverse events were reported.

**Conclusions:**

The study results give strong support for the feasibility of a full-scale trial. Both arms had high rates of recruitment, retention, and acceptability. There was promising support of the benefits of integrated cognitive-behavioural therapy-based vestibular rehabilitation compared to gold standard vestibular rehabilitation. The study fulfilled all the a-priori criteria to advance to a full-scale efficacy trial.

**Trial registration number:**

ISRCTN10420559.

## Introduction

Persistent Postural Perceptual Dizziness (PPPD) is a complex functional neuro-vestibular disorder characterised by persistent dizziness, non-spinning vertigo and/or unsteadiness [37]. It is thought to be a long-term maladaptation to neuro-otological, neurological or medical illness, and/or psychological distress. Since its international classification by the Bárány Society [37], PPPD is increasingly recognised as the single most common vestibular syndrome in specialised outpatient clinics [38] and likely represents the vast majority of patients referred to vestibular rehabilitation [36]. People living with PPPD have poor quality of life, severe dizziness handicap and an elevated risk of anxiety and depression [3, 40].

Tailored treatment strategies have been recommended, including pharmacotherapy with selective serotonin reuptake inhibitors (SSRI), physiotherapy (vestibular rehabilitation) and cognitive-behavioural therapy (CBT), but there is a lack of prospective, randomised controlled trials or information on prognosis or outcomes [30]. Vestibular rehabilitation therapy (VRT) is an established exercise-based treatment for people with structural vestibular disorders [26] that is usually recommended for people with PPPD [27, 41]. However, the exercises must be carefully graded to avoid intolerable symptom provocation and psychological factors are known to negatively affect outcome [42]. There is limited evidence in favour of CBT in PPPD [10], although one study reported short-term relief [20]. However, there are better results when CBT is adapted to target illness-specific factors such as anxiety-related postural behaviour [5]. There are also promising multidisciplinary programs [2, 25], but these can be costly and difficult to replicate. Due to their similarities, there has been a desire to combine CBT and VRT for a long time [4, 36, 44], but no theory-driven, evidence-based intervention with a standardised treatment manual currently exists. To date there are only a few case reports and pilot studies [1, 22–24, 31]. Moreover, previous trials do not test interventions against current best practice.

To address this gap, we developed a combined CBT-VRT intervention based on existing research data and theoretical modelling of the psychological factors that contribute to dizziness handicap [17, 18]. Based on those findings and working in partnership with patient representatives, we developed a patient manual. We believe there is a better chance of acceptability and success when the intervention can remediate specific perpetuators of dizziness and be integrated within a physiotherapy programme.

The aim of this study was to evaluate the feasibility and acceptability of the integrated CBT and VRT (INVEST) intervention and trial methodology, for people with PPPD, as part of the preparation for a full-scale randomized controlled trial. Specific objectives were to determine the recruitment and retention rate, to test the utility of a range of outcome measures, levels of acceptability, assess adherence and to collect outcome data to explore treatment effects and estimate key elements that would inform a large-scale trial. For a breakdown of the detailed study objectives and predefined progression criteria, please see the published protocol [16].

## Methods

### Design

Two-armed parallel groups randomised controlled single centre feasibility trial with online assessment before randomisation (T0) and at follow-up four months post-randomisation (T1). Participants in the INVEST arm were also invited to participate in a qualitative interview after T1 (results will be reported elsewhere). There were no changes from the published research protocol, which contains more detailed methods and intervention specifics [16].

### Setting

An outpatient tertiary (specialist) setting at St George’s University NHS Foundation Trust in urban London, United Kingdom. Recruitment was between November 2020 and August 2021 but was discontinuous due to the status of clinics during the COVID-19 pandemic.

### Participants

Adults (aged 18 or older) with persistent movement triggered dizziness for ≥ 3 months due to a vestibular diagnosis (according to the international classification of vestibular disorders), scoring ≥ 40 on the Dizziness Handicap Inventory (DHI), able to read and speak English, and willing and able to take part in the study were eligible.

Patients were excluded if they had another active condition which could interfere with their ability to participate in physiotherapy, including ≥ 3 headache/migraines a month, severe mental health disorder, another neurological disorder, acute orthopaedic disorders affecting balance and gait, and active Meniere’s disease or benign paroxysmal positional vertigo (BPPV). We also excluded patients with central (such as strokes, intracranial tumours, degenerative disorders and metabolic conditions, but not including functional dizziness/PPPD or vestibular migraine) [7] or bilateral vestibulopathy (according to Barany criteria [39]).

Participants were identified by audio-vestibular physicians and/or on referral to the vestibular physiotherapy department. Pre-screening excluded patients with active BPPV or unrelated audio-vestibular disorders that do not require vestibular physiotherapy. Potential participants were screened for eligibility via telephone and sent a participant information sheet by email or post according to their preference. Participants enrolled by completing an online consent form. No compensation was provided for taking part.

### Sample size determination

The intended sample size was 40 assuming participation rates of 33% and drop-out rates of 20%, to estimate 95% confidence intervals for the participation and drop-out rates within a maximum interval of ± 9% and ± 16% respectively.

### Randomisation

The random allocation sequence was generated using a minimisation procedure with a probability of 0.8 to assure similar distribution of selected participant factors between trial groups, which included three dichotomous outcomes: gender (male/female), age (18–60/over 60) and dizziness handicap (DHI score 40–59/ ≥ 60). Participants were randomized consecutively in the order in which they were referred to the study, and all staff and patients were blinded to allocation sequence. Randomisation was implemented independently by King’s Clinical Trials Unit via an online electronic system.

### Interventions

The interventions are detailed in the protocol [16]. Each arm was delivered by a different senior specialist grade physiotherapist (DH and KF).

#### INVEST

In brief, INVEST included six-sessions of individual CBT-informed VRT aimed specifically at dizziness (not depression or anxiety) with a patient manual and therapist support. The initial session was 60 min, follow-up appointments were 30 min, and all were led by the same physiotherapist (author DH) who had additional training in CBT. There was a focus on transparency in communication which started with a shared cognitive-behavioural formulation and psychoeducation. Exercises were customised and focussed on normalising any maladaptive postural strategies (e.g., ‘high-threat’ postural control) early on, and habituation. Exercises were performed in clinic and at home. Other techniques included goal setting, activity planning and graded exercise, attention allocation and relaxation techniques, cognitive therapy focussed on illness beliefs, exposure in-vivo with behavioural experiments for dizziness related fear, relapse management and prevention.

##### Vestibular rehabilitation (control)

The six-sessions of individual VRT were time-matched to the INVEST protocol. The VRT represented ‘gold standard’ treatment based on evidence-based Clinical Practice Guidelines [14] and recommendations for people with PPPD [30] to promote graded habituation to movement and visual stimuli. Participants were provided with a customised exercise programme, performed in clinic and at home, which included a range of general exercises (e.g., walking programmes) and more specific adaptation, habituation, visual desensitisation, static and dynamic balance exercises.

### Measures

#### Sociodemographic and clinical data

Self-reported sociodemographic data were collected at baseline. Clinical data were extracted from medical records at T0 and T1. A diagnosis of PPPD was based on the latest Barany classification [37]. Since it is common for people with PPPD to have other vestibular disorders or conditions which provoke dizziness, relevant co-existing conditions were extracted from each participant’s medical records. Results of any vestibular laboratory function testing were also extracted and interpreted according to their respective normative values.

#### Feasibility outcomes

Numbers of eligible people recruited, willingness to be randomised and retention rates were collected. Acceptability was evaluated at follow-up using an eight-item scale to assess the constructs in the theoretical framework of acceptability [33].

#### Self-report outcomes

Participants completed all self-report measures online at T0 and T1 independently at home including measures of Dizziness Handicap (DHI) [21], visually induced dizziness (Visual Vertigo Analogue Scale [VVAS]) [8], dizziness interference (percentage of time symptoms interfere with life [%TSI]) [13] and health status (European Quality of Life questionnaire [EQ5D]) [12]. All scales are previously well-validated in people with chronic dizziness (see protocol for details) [16].

Putative process variables measured included negative dizziness specific illness perceptions (Brief Illness Perception Questionnaire [B-IPQ]) [6], cognitive and behavioural responses to dizziness (Cognitive and Behavioural Responses to Symptoms Questionnaire [CBRQ]) [29], depression (Patient Health Questionnaire-9 [PHQ-9]) [34], anxiety (Generalized Anxiety Disorders-7 [GAD-7]) [35] and combined distress (Patient Health Questionnaire Anxiety and Depression Scale [PHQ-ADS]) [19]. Internal consistency for all outcome measures was acceptable (Cronbach alpha all ≥ 0.7).

At T1, participants were asked to self-report any other new treatments started during the study and a record of any adverse events was updated throughout.

#### Balance

All participants completed either the mini–Balance Evaluation Systems Test (mini-BESTest) [11] or a hybrid balance assessment if conducted virtually at T0 and T1. The hybrid balance assessment consisted of the Mini-BESTest excluding those items that were not possible to conduct virtually. The most difficult item to measure was reactive postural control since this requires a therapist to provide an external perturbation. To account for this missing data, patients were dichotomised as demonstrating either normal or abnormal balance control based on the available data and therapist judgement.

### Statistical analysis

Questionnaires were completed online and there was no question item missing data. Descriptive statistics were used to summarise the number of patients approached, screened, eligible, consented, and randomised. Reasons for non-consent, exclusion, and drop-out, at each stage of the study, were recorded. Similarly, descriptive statistics were computed to report adherence to the intervention.

Internal consistency of the measures was assessed using the Cronbach alpha coefficient at both T0 and T1. The EQ5D health state utility score was calculated from individual health profiles using the value set for England [9]. Mean and standard deviations (SD) are provided for all self-report outcomes by visit and by treatment. Estimates of treatment effect at T1 were based on an analysis of covariance (ANCOVA) to estimate the postintervention mean difference. The analysis adjusted for the baseline level of the outcome variable, baseline DHI, age and sex. Group allocation was included as an indicator variable following the intention-to-treat principle. Given the feasibility nature of the trial, with a small sample size not powered to detect between group differences, the statistical significance of any post-randomisation group differences was not assessed; instead, effect sizes were calculated as standardised mean differences using Hedge’s g (SMDg) applying the small sample bias correction factor [15].

## Results

### Participant flow and feasibility outcomes

**Fig. 1:**
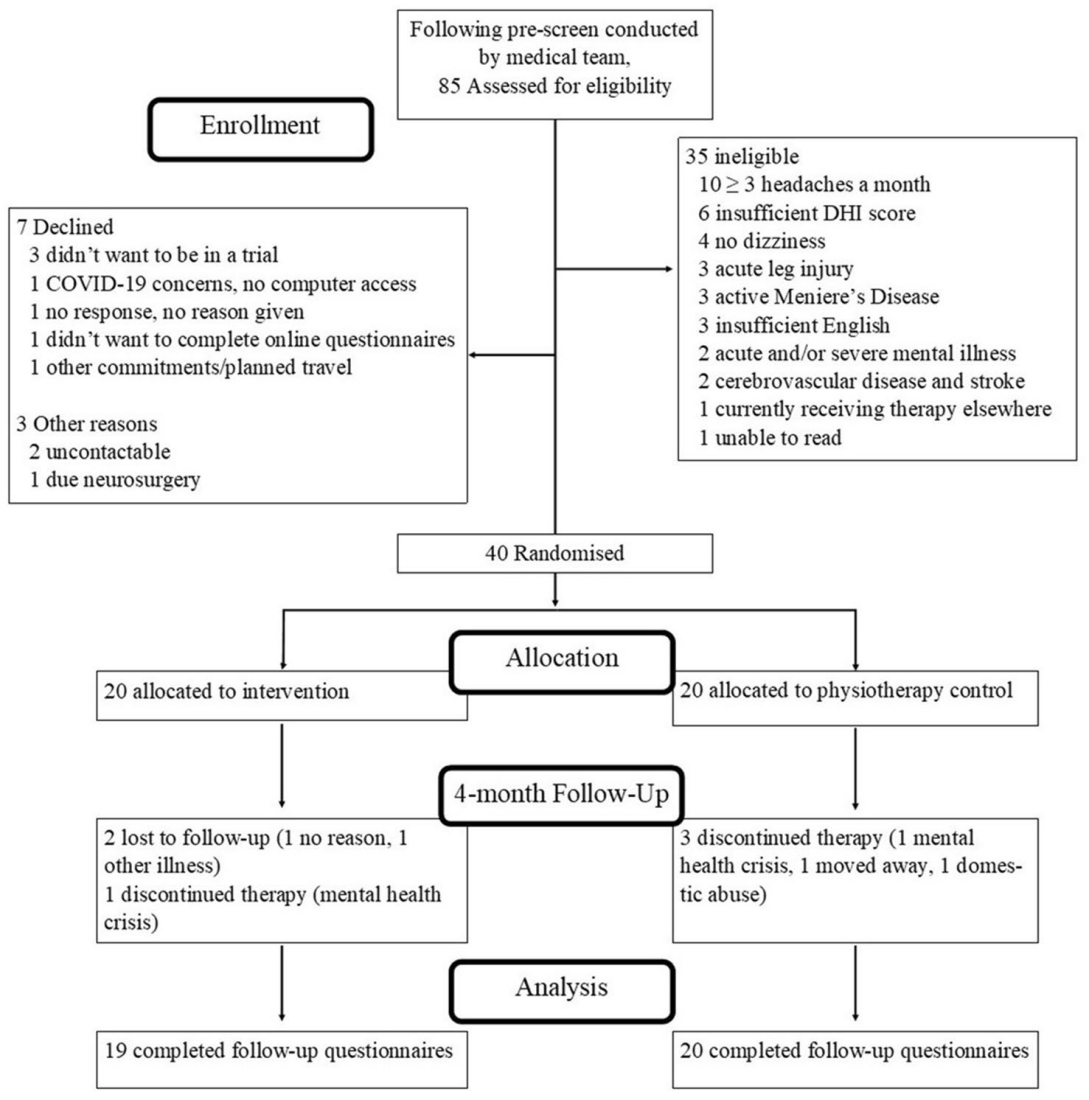
Participant flow diagram. *DHI* dizziness handicap inventory

Participant flow is shown in Fig. 1. After a pre-screen conducted by the medical team, 85 people were approached and 35 (41%; 95% CI 31%–52%) excluded due to ineligibility (reasons in Fig. 1). Seven (14%; 95% CI 6%–26%) eligible patients did not want to be randomised, three (6%) because of concern about being in a trial. Another three patients were not recruited as they were untraceable or unavailable after initial screening. Forty out of 50 eligible participants (80% enrolment rate; 95% CI 66–90%) were recruited and randomly assigned to the INVEST intervention (*n* = 20) or gold standard VRT (control; *n* = 20).

Drop-out rate from INVEST was 15% (95% CI 3–38%) and 20% for VRT (6–44%). One participant lost to follow-up did not complete the follow-up questionnaires (trial drop-out 2.5%; 95% CI < 1 to 13%).

### Baseline characteristics

Table 1 shows baseline characteristics by group. The groups were equally distributed for age, sex, sociodemographic, clinical (including dizziness handicap), and psychological characteristics at baseline. The mean age was 44.5 years (SD = 17, range 19–79) and 32/40 (80%) were women. Median symptom duration was 2 years (IQR 46.5 months; range 5 months to 21 years).

**Table 1:** Baseline characteristics

	Group allocation	
	INVEST	VRT
Age (mean, SD)	44.60 (16.96)	44.30 (17.44)
Sex (*n*, %)		
Female	16 (80%)	16 (80%)
Male	4 (20%)	4 (20%)
Ethnicity (*n*, %)		
White	13 (65%)	15 (75%)
Mixed or multiple ethnic groups	1 (5%)	1 (5%)
Asian or Asian British	2 (10%)	2 (10%)
Black, African, Caribbean, or Black British	4 (20%)	1 (5%)
Other ethnic group	0 (0%)	1 (5%)
Education (*n*, %)		
Higher education	13 (65%)	12 (60%)
College, vocational level 3, and equivalents	3 (15%)	5 (25%)
High school, vocational level 2, and equivalents	0 (0%)	2 (10%)
Qualifications at level 1 and below	1 (5%)	0 (0%)
Other qualifications: level unknown	1 (5%)	0 (0%)
No qualifications	2 (10%)	1 (5%)
Employment status (*n*, %)		
Employed	13 (65%)	10 (50%)
Unemployed	4 (20%)	7 (35%)
Student	1 (5%)	2 (10%)
Retired	2 (10%)	1 (5%)
Clinical variables		
Diagnosis (*n*, %)		
Persistent postural perceptual dizziness	20 (100%)	20(100%)
Illness duration, months (median, IQR)	24 (95)	21 (32)
Another related condition/trigger (*n*, %)		
Vestibular migraine	9 (45%)	8 (40%)
Clinical features of anxiety	9 (45%)	5 (25%)
Unilateral peripheral vestibulopathy	5 (25%)	8 (40%)
BPPV	2 (10%)	6 (30%)
Meniere’s/migraine overlap	0 (0%)	1 (5%)
Meniere’s disease	1 (5%)	0 (0%)
Vestibular testing abnormalities (*n*, %)		
Unilateral vestibular dysfunction	6 (30%)	2 (10%)
Normal vestibular function testing	11 (55%)	11 (55%)
On SSRI/SNRI medication (*n*, %)	1 (5%)	2 (10%)
Dizziness handicap Inventory (mean, SD)	63.80 (17.84)	65.10 (14.76)

*SD* standard deviation, *IQR* inter-quartile range, *BPPV* benign paroxysmal positional vertigo, *SSRI* selective serotonin reuptake inhibitors, *SNRI* serotonin-norepinephrine reuptake inhibitor, *VRT* vestibular rehabilitation

### Acceptability

**Fig. 2:**
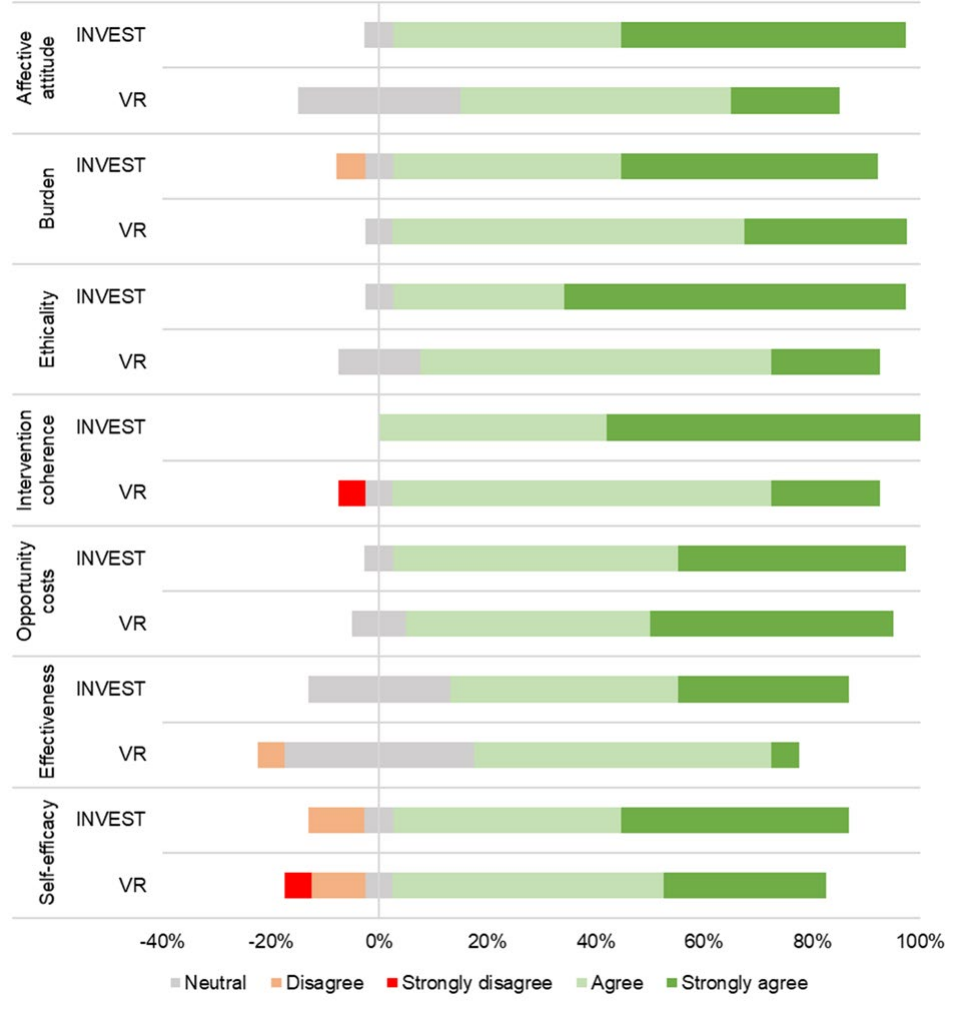
Likert scale acceptability data according to group allocation. *INVEST* integrated intervention, *VRT* gold standard vestibular rehabilitation

Figure 2 shows responses to the acceptability questionnaire. More than 80% of participants in both arms rated ‘agree’ or ‘strongly agree’ in favour for each domain. Participants in the INVEST arm tended to have slightly stronger positive opinions compared to the control arm.

### Outcomes

Twelve participants (60%; 95% CI 36–81%) in the intervention compared to seven (35%; 95% CI 15–59%) in the control group achieved a reliable improvement according to the dizziness handicap (> 18 point reduction). Nine participants (45%; 95% CI 23–68%) in the intervention had a ‘reliable recovery’ as defined by a DHI score below 30, compared to one (5%; 95% CI < 1 to 25%) in the control group.

**Fig. 3:**
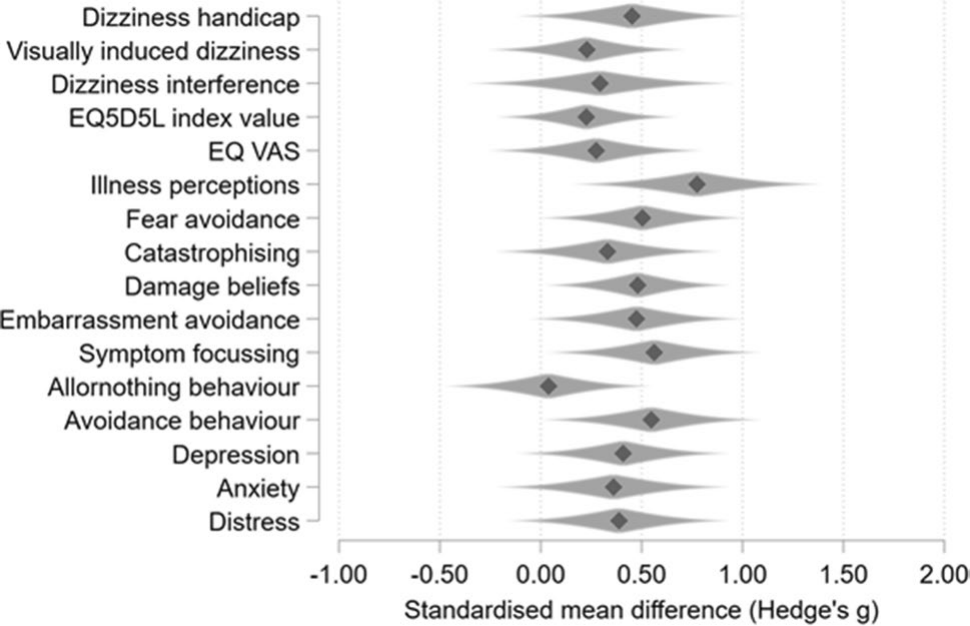
Treatment effect sizes and confidence intervals

Table 2 provides prescores and postcores for the self-report questionnaires and estimates of treatment effect at T1 adjusted for baseline levels, dizziness handicap, age, and sex. Figure 3 shows a forest plot with confidence intervals to visualise the estimates of the treatment effects and their uncertainty. On average, all outcomes improved from baseline for both groups. Between-group differences at T1, adjusting for baseline level, typically demonstrated small to moderate effects in favour of INVEST for all dizziness and qualtiy of life related outcomes. In terms of putative mechanisms, reductions in negative illness perceptions showed the largest effect (SMDg 0.77) with moderate effects on distress and almost all symptom interpretation variables suggested greater reductions in catastrophising, beliefs that symptoms cause damage, and embarresment and fear avoidance. INVEST did not appear to have greater benefit for all-or-nothing behaviour (SMDg 0.04).

**Table 2:** Means of outcome measures at each assessment and post-randomisation treatment effects

Outcome measure	INVEST intervention		VRT physiotherapy control		Adjusted mean difference^a^		
	Baseline (*n* = 20) Mean (SD)	Follow-up (*n* = 19) Mean (SD)	Baseline (*n* = 20) Mean (SD)	Follow-up (*n* = 20) Mean (SD)	Mean difference (SE)	95% CI for difference	Hedge’s *g* (95% CI)
Dizziness handicap							
DHI	63.80 (17.84)	37.16 (23.84)	65.10 (14.76)	48.80 (19.44)	10.04 (6.48)	− 3.14 to 23.21	0.45 (− 0.12, 1.02)
Visually induced dizziness							
VVAS	54.33 (20.97)	30.41 (24.29)	54.44 (20.47)	38.33 (22.45)	5.45 (5.90)	− 6.557 to 17.46	0.23 (− 0.26, 0.71)
Dizziness interference							
%TSI	57.00 (29.80)	29.32 (26.08)	65.50 (27.32)	39.70 (27.64)	8.05 (9.12)	− 10.50 to 26.60	0.29 (− 0.36, 0.95)
Health state							
EQ-5D-5L index value	0.52 (0.25)	0.67 (0.27)	0.50 (0.26)	0.58 (0.25)	− 0.06 (0.06)	− 0.19 to 0.07	0.23 (− 0.22, 0.67)
EQ VAS	47.75 (23.33)	57.79 (24.30)	48.90 (26.58)	50.45 (24.57)	− 6.85 (6.71)	− 20.50 to 6.80	0.27 (− 0.25, 0.80)
Negative dizziness perceptions							
B-IPQ	55.75 (10.78)	32.79 (15.39)	57.40 (7.37)	46.20 (14.27)	11.73 (4.75)	2.07 to 21.39	0.77 (0.16, 1.39)
CBRQ domains							
Fear avoidance	14.45 (4.37)	8.74 (4.59)	15.35 (4.67)	11.55 (4.51)	2.34 (1.21)	− 0.13 to 4.81	0.50 (− 0.01, 1.01)
Catastrophising	9.30 (3.25)	5.42 (4.25)	10.30 (3.26)	7.60 (3.41)	1.30 (1.11)	− 0.955 to 3.56	0.33 (− 0.22, 0.88)
Damage beliefs	11.95 (3.32)	7.53 (4.77)	12.15 (3.28)	9.80 (3.59)	2.07 (1.01)	0.14 to 4.13	0.48 (0.02, 0.94)
Embarrassment avoidance	14.65 (5.48)	8.58 (6.70)	14.05 (5.35)	10.80 (4.80)	2.82 (1.60)	− 0.44 to 6.07	0.47 (− 0.05, 1.00)
Symptom focussing	16.40 (4.86)	10.84 (5.47)	17.80 (4.46)	14.70 (4.79)	2.95 (1.41)	0.08 to 5.81	0.56 (0.04, 1.09)
All-or-nothing behaviour	8.50 (4.40)	6.53 (4.41)	7.85 (4.90)	6.45 (4.17)	0.17 (1.14)	− 2.14 to 2.48	0.04 (− 0.47, 0.55)
Rest/Avoidance behaviour	14.75 (6.79)	8.16 (5.96)	13.25 (6.63)	10.35 (5.02)	3.08 (1.56)	− 0.10 to 6.26	0.55 (0.00, 1.09)
Depression							
PHQ9	10.35 (6.10)	5.37 (5.06)	12.50 (8.07)	9.65 (7.32)	2.62 (1.70)	− 0.84 to 6.08	0.41 (− 0.11, 0.93)
Anxiety							
GAD7	7.10 (4.95)	4.47 (4.12)	10.40 (6.992)	8.30 (6.58)	2.02 (1.64)	− 1.32 to 5.35	0.36 (− 0.21, 0.93)
Distress							
PHQ-ADS	17.45 (10.39)	9.84 (8.90)	22.90 (14.47)	17.95 (13.47)	4.52 (3.24)	− 2.08 to 11.11	0.39 (− 0.16, 0.93)

*VRT* vestibular rehabilitation, *DHI* dizziness handicap inventory, *VVAS* visual vertigo analogue scale, *%TSI* Percentage time symptoms interfere with normal activities, *EG-5D-5L* European quality of life questionnaire (EuroQol), *EQ VAS* EuroQol visual analogue scale, *B-IPQ* brief illness perceptions questionnaire, *CBRQ* cognitive behavioural responses to symptoms questionnaire, *PHQ9* patient health questionnaire 9 item scale, *GAD7* generalised anxiety disorders 7 item scale, *PHQ-ADS* patient health questionnaire-anxiety and depression scale

^a^Adjustment for multiple comparisons: Bonferroni

At baseline (T0), 16 participants (80%; 95% CI 56–94%) in the intervention group and 17 participants (85%; 95% CI 62–97%) in the control group were identified to have a abnormal balance scores. At follow-up only one participant (5%; 95% CI < 1 to 25%) in the intervention group and five participants (25%; 95% CI 9–49%) in the control group were identified to still have abnormal balance. Thirty-three participants completed the mini-Bestest at baseline (intervention mean 23.1, SD 3.57; control mean 24, SD 3.56) and 25 participants completed it at T1 (intervention mean 27.6, SD 1.12; control mean 26.9, SD 1.85).

### Adherence to INVEST

All participants completed the first two sessions. Eighty percent (*n* = 16; 95% CI 56–94%) completed all six sessions. One participant missed session six due to other commitments. Two dropped out after the second session and one dropped out after 3 sessions. Seventy-five percent of sessions were conducted in person and 25% remotely. Most sessions adhered to the prescribed duration except for exposure in-vivo (usually session 3) which usually lasted 45 min. One participant from each group had a relapse of BPPV (benign paroxysmal positional vertigo) which was successfully treated with a single canalith repositioning procedure. Table 3 lists other treatments started during the trial.

**Table 3:** Other treatments started during the trial and any adverse events

	INVEST	VRT
Other treatments started during trial (*n*)		
Amitriptyline/nortriptyline	0	2
SSRI/SNRI	1	1
Talking therapies	0	2
Betahistine	0	1
Fertility treatment	1	0
Pelvic adhesiolysis	1	0
Physiotherapy for pain condition	1	1
Herbal supplements	1	0
Adverse events (*n*)		
BPPV relapse	1	1
Migraine flare	1	1
Mental illness	1	1
Injurious fall, not related to exercise	1	0
Traumatic family event	1	0
Victim of domestic abuse	0	1
Hospital admission, unrelated condition	0	1

*VRT* gold-standard vestibular rehabilitation

### Adverse events

Table 3 lists adverse events for each group. One participant from each group had exacerbation of migraines which could reasonably be attributed to exercise. Otherwise, participants did not attribute any other adverse incident to intervention-related activity.

## Discussion

This study aimed to establish the feasibility and acceptability of a large RCT and potential benefits of a theory-based CBT-informed VRT intervention when compared to current gold-standard VRT for people with PPPD. The study met all the a-priori criteria to progress to a full-scale efficacy trial, including 80% of eligible patients participating (pre-defined criteria > 70%), 15% therapy and 2.5% trial drop-out rates (criteria < 20%), comparable acceptability ratings to current gold standard VRT, and 80% adherence to sessions (criteria > 60%). Fifty-nine percent of patients screened met the selection criteria and the enrolment rate was 80%. This translates to roughly two patients screened for every one participant. Given the high prevalence of PPPD in audio-vestibular, neuro-otology, and VRT clinics there are sufficient patients to run a fully powered RCT. High rates of recruitment and retention point to an INVEST RCT being acceptable.

According to the acceptability survey and exploratory treatment effect sizes, the intervention appeared to be both acceptable and beneficial. All treatment effects favoured the INVEST intervention. Treatment effects for dizziness handicap were clinically meaningful and a larger proportion of the intervention group achieved a reliable improvement (60%) vs. the control group (35%). Although these treatment effects cannot be taken as evidence for efficacy, they compare favourably to similar published studies [1, 22–24, 31]. However, given the small sample size uncertainty in these estimates was considerable. The findings still provide a strong signal for efficacy that supports the justification for a full-scale efficacy study. Participants in this current study had a high level of dizziness handicap and a median illness duration of 2 years, which is usually associated with a poor prognosis [42], indicating that these may be important factors to consider as treatment effect modifiers in a full-scale trial. Since treatment effects were not universal, we believe there is further scope for the intervention to be improved. Suggestions for some of the improvements will be presented in the detailed qualitative analysis to follow.

Between-group comparison for putative process variables suggests that INVEST is changing the proposed mechanisms of action as intended. This is particularly true for negative beliefs about dizziness and the way in which patients attend to and appraise dizziness as threatening or embarrassing. Reductions in avoidance and resting in response to symptoms was also greater in INVEST. It was surprising that all-or-nothing behaviour did not show a larger treatment effect, since our previous prospective data found this to be a strong predictor and was a predominant feature of INVEST [17, 18]. Both groups improved so this may also reflect similarities between the interventions in terms of pacing and graded exercise.

The gait and balance outcome measures were sometimes difficult to execute when completed over video. The Mini-BESTest was suboptimal since it is only validated for face-to-face evaluation. Further, many people with PPPD exhibit features of ‘functional gait disorder’ [32]. As discussed by Nicholson et al. [28], the unique clinical aspects of functional disorders means that the usual prioritization of ‘objective’ or ‘subjective’ measures may not be appropriate when it comes to measuring balance and gait. For example, due to temporal variability in balance performance, objective snapshot tests such as gait speed may not accurately reflect the general state of the disorder. Likewise, since attention, and therefore clinical examination, can modify gait performance in people with PPPD, clinical assessment may not reflect actual performance outside of this context. Objective measures such as posturography have shown merit in PPPD although again this requires face-to-face evaluation, and the cost is a significant barrier. The advent of wearable motion sensors may be a useful compromise and other clinical tests of dynamic gait performance may be more practical. INVEST did appear to simultaneously improve postural control, and a dichotomised outcome has been adopted in other studies [31], but the lack of blinding and validity is a limitation. Improvements in balance observed in the INVEST group could be because the balance exercises were focussed on allocating attention away from consciously controlling balance and fear driven adaptations to balance control [43], so a measure that could reliably evaluate this would be preferable.

There were no reported serious adverse incidents attributed to the intervention. The risk profile appears similar to standard vestibular rehabilitation. There was a single mental health-related adverse event in both groups, which both patients attributed to external factors rather than to trial interventions. There were no adverse reactions to any behavioural experiment. Other social external traumatic events occurred, which may reflect the presence of social risk factors associated with persistent functional symptoms. Interestingly, one participant from each group also had a reoccurrence of BPPV. This provides another benefit of such an intervention being delivered by a physiotherapist or multidisciplinary team, because such conditions can be easily identified and treated quickly, minimising the impact of symptom relapse.

Other limitations in our study must be noted. This was a single site RCT, and the first author, who led the INVEST development, was the physiotherapist delivering it in this trial. To try and counteract this, the person delivering the standard VRT arm was also a senior physiotherapist specialising in VRT. A full-scale multi-centre efficacy trial will need to consider the level of training and supervision required for a range of physiotherapists to deliver it successfully. We did not use a standardised diagnostic schedule to ascertain clinically significant psychiatric comorbidity as a basis for study exclusion. This may have led to inclusion of inappropriate patients, particularly people with post-traumatic stress disorder who require specialist CBT programmes. For practical purposes during the pandemic, we allowed flexibility in the mode of delivery between face-to-face and virtual appointments. Whilst this likely reflects the way services will continue to operate, there is a lack of evidence to say if this affects outcomes and some participants had a strong perception that face-to -face was better. Therefore, future studies may need to control for delivery mode. Our control group represented current gold VRT, although we suspect there may have also been some treatment contamination as both therapists worked in the same department. Future trials will need to consider ways to reduce such contamination, such as cluster randomisation, or spatially separating trial arms. As with all behavioural trials, participants and therapists could not be blinded to treatment group, which may have introduced bias. However, the trial information provided made it clear both were treatments for PPPD with no expectation that one was better than the other. Likewise, it is difficult to tightly control the therapy being delivered when the treatment requires a tailored approach. Using the patient manual was one such approach, although digitalising aspects of the intervention remains an option in the future. Sessions were also audiotaped for supervision and fidelity purposes. More work is needed to ensure fidelity of the standard care arm in a larger trial. Most outcomes were subjective patient-reported outcomes completed online, which may be influenced by many factors, including lack of blinding. However, we argue that it is impractical and illogical to construct a placebo therapy which would contain the same characteristics as the treatment for which it serves as a control. Instead, as was a strength of this study, comparison to a current gold standard therapy should be used when evaluating treatment efficacy and that the only person that needs to be blinded is the statistician.

## Conclusions

Preliminary trial findings support the acceptability and feasibility of INVEST, a CBT-informed VRT intervention aimed at dizziness for people with PPPD. Estimates support medium treatment effects and potential benefits compared to gold standard VRT in a small group of patients that have high levels of dizziness related disability and a poor prognosis with the current available treatment. Findings strongly support the need for a multicentre randomised trial of the INVEST intervention.

## Data Availability

Data will be made available upon reasonable request to the corresponding author.

## Declarations

### Trial registration

ISRCTN10420559.

### Study protocol location

10.1186/s40814-021-00896.
